# Tumor suppressive role of mitochondrial sirtuin 4 in induction of G2/M cell cycle arrest and apoptosis in hepatitis B virus-related hepatocellular carcinoma

**DOI:** 10.1038/s41420-021-00470-8

**Published:** 2021-04-30

**Authors:** Fung-Yu Huang, Danny Ka-Ho Wong, Wai-Kay Seto, Lung-Yi Mak, Tan-To Cheung, Man-Fung Yuen

**Affiliations:** 1 Department of Medicine, The University of Hong Kong, Queen Mary Hospital, Hong Kong SAR, China; 2 State Key Laboratory of Liver Research, The University of Hong Kong, Hong Kong SAR, China; 3 Department of Surgery, The University of Hong Kong, Queen Mary Hospital, Hong Kong SAR, China

**Keywords:** Oncogenesis, Cancer

## Abstract

Hepatocellular carcinoma (HCC) is developed from uncontrolled cell growth after the malignant transformation of hepatocytes. The hepatitis B virus (HBV) X protein (HBx) has shown to induce cell cycle progression and hepatocarcinogenesis. A sub-fraction of HBx is localized in the mitochondria. Sirtuin 4 (SIRT4), a mitochondrial protein, has been demonstrated to play a tumor-suppressive role in many cancers, including HCC. However, little is known about the association between mitochondrial HBx and SIRT4 during hepatocarcinogenesis. We aimed to investigate the clinical significance and functional role of SIRT4 in HBV-related HCC. SIRT4 expression was significantly lower in the HCC tissues collected from 30 patients with HBV-related HCC than in normal liver tissues from control patients (*p* < 0.0001). TCGA data analysis indicated that SIRT4 expression was also lower in patients with HBV infection than in those without, and SIRT4 levels were positively associated with better patient survival. Similarly, HCC cell lines had lower SIRT4 expression than normal liver cell lines (all *p* < 0.01). Among the HCC cell lines, those harbored HBV had a lower SIRT4 expression than those without HBV (*p* < 0.0001). In vitro experiments revealed that stable HBx transfection suppressed SIRT4 expression in both HepG2 and Huh7 cells (both *p* < 0.001). Ectopic SIRT4 overexpression alone could induce cellular senescence through arresting cell-cycle progression at G2/M, and inducing cell apoptosis in HCC cells. Mechanistically, SIRT4 upregulated cell-cycle governing genes p16 and p21 protein expression, suppressed CyclinB1/Cdc2 and Cdc25c which normally induce cell-cycle progression, and suppressed survivin to induce apoptosis. Our findings demonstrate the interaction between HBV and SIRT4 in the context of HCC. SIRT4 involves in G2/M DNA damage checkpoint control and genomic stability in hepatocarcinogenesis, which could be targeted for future anticancer strategies.

## Introduction

Hepatocellular carcinoma (HCC) is a leading cause of cancer-related mortality owing to late symptom manifestations and limited treatment options^1^. Chronic hepatitis B virus (HBV) infection is a predominant risk factor for HCC and accounts for 50–80% of HCC cases worldwide^2^. However, the complete pathogenic mechanisms by which HBV contributes to the development of HCC remains largely unknown^3–5^.

HBV DNA can randomly integrate into the host hepatocyte genome during chronic infection^6^. The HBV genome contains four genes, namely the core/precore, PreS/S, polymerase, and X genes, among which only the X gene is well preserved in the integrated HBV DNA in the host genome of HCC patients^7^. The HBV X protein (HBx) is believed to play a prominent role in hepatocarcinogenesis^8–10^. Previous studies suggest that HBx contributes to the progression of HCC by interacting with cellular proteins and altering many signaling processes including transcription regulation, signal transduction, cell-cycle progression, cellular senescence, apoptosis, and protein degradation^8,11–14^. Owing to the fact that treatment options against HCC are limited, targeting the HBx-altered molecular networks might offer new therapeutic options for early detection and treatment of this disease.

Mitochondria are vital organelles that regulate a number of important cellular processes such as apoptosis, metabolism, and signal transduction^15^. Although HBx is predominantly a cytoplasmic protein, a sub-fraction of cytosolic HBx has been found inside the mitochondria, suggesting that HBx may manipulate mitochondrial functions^16,17^. Indeed, several studies have demonstrated that HBx affects mitochondrial physiology by downregulating mitochondrial enzyme expression and causing an accumulation of reactive oxygen species in the mitochondria^18–20^. Induction of oxidative stress is often associated with liver inflammation and DNA damage, both of which are known pathways of HBV-induced hepatocarcinogenesis^21^. Therefore, identification and characterization of host factors that interact with mitochondrial HBx will provide insights into the pathogenesis of HBV-related HCC and hopefully facilitate the development of anti-HCC therapeutics.

Sirtuins are a class of proteins that are important in maintaining cellular homeostasis. They play important roles in various cellular processes, including cellular apoptosis, metabolism, stress resistance, and genomic stability^22^. Mammalian sirtuins consist of seven members, namely sirtuins 1–7 (SIRT 1–7). Of these, the nuclear sirtuins, SIRT1, SIRT6, and SIRT7, as well as the cytosolic SIRT2, are well-studied with respect to their roles in regulating inflammatory responses^23^. Comparatively, the mitochondrial sirtuins (SIRT 3–5) are less studied. Recent data suggest that mitochondrial sirtuins protect against the development of age-related chronic diseases^24^. Among the three mitochondrial sirtuins, SIRT4 is the least studied member^25^. Despite this, there is increasing evidence suggesting that SIRT4 acts as a mitochondrial-localized tumor suppressor in various cancers such as breast cancer, prostate cancer, esophageal squamous cell carcinoma, and colorectal cancer^26–30^. The tumor-suppressive role of SIRT4 is also implicated in HCC^31,32^. However, the role of SIRT4 as a tumor suppressor in the setting of HBV-induced HCC has not been explored. Based on the role of mitochondrial HBx in inducing oxidative stress and the tumor-suppressive function of SIRT4, both of which share the same mitochondrial localization, this study aimed to investigate the functional role of SIRT4 and its interaction with HBx in the development of HBV-related HCC.

## Results

### Patient characteristics

Clinicopathological characteristics of the 30 patients with HBV-related HCC are summarized in Supplementary Table S2. There were 24 males and 6 females, with a median age of 55.4 years (range: 36.3–76). Twenty-five patients (83.3%) had advanced fibrosis or cirrhosis in the adjacent non-tumor tissues. Twenty-one (70.0%) patients were at TNM stage I and II and 9 (30.0%) patients at stage III. Twenty-five patients (83.3%) had well-to-moderate differentiated tumors. Tumor invasion and tumor encapsulation were seen in 16 (53.3%) and 20 (66.7%) patients, respectively.

### SIRT4 expression in human HCC and its association with patient survival

**Fig. 1:**
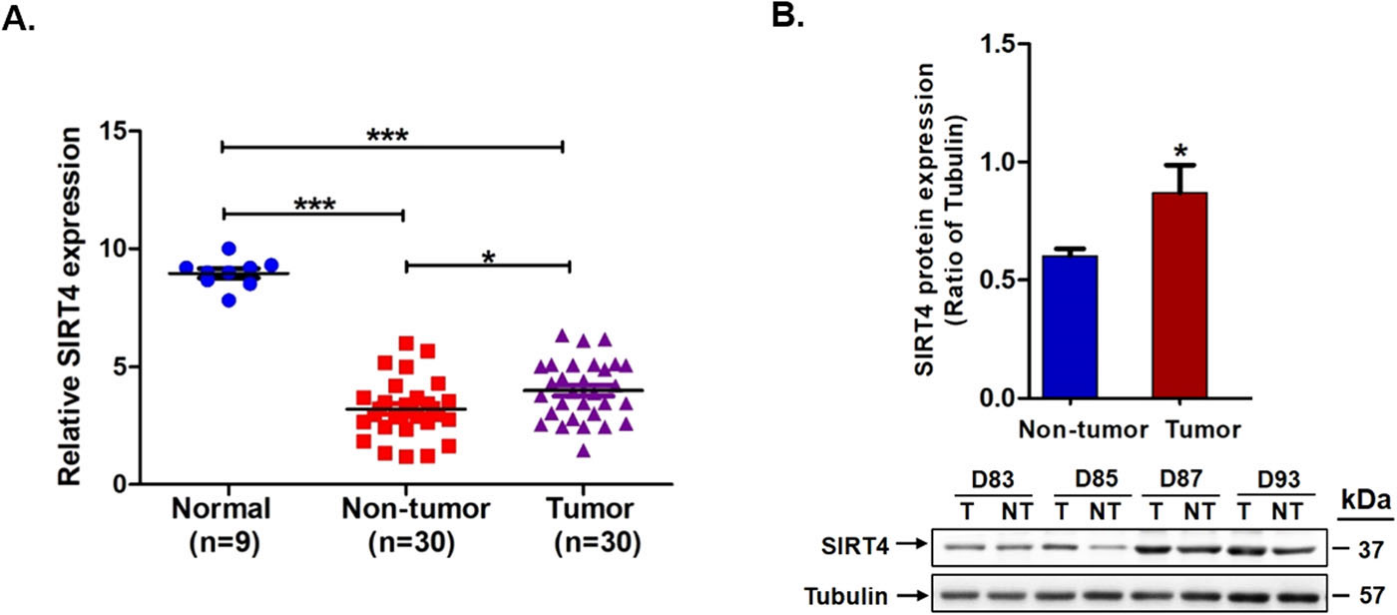
The expression of SIRT4 was downregulated in HBV-related HCC tissues. **A** qPCR analysis on mRNA expression of SIRT4 in 9 normal liver biopsies and 30 pairs of HCC tumor and their adjacent non-tumor tissues. **B** SIRT4 protein expression was slightly increased in HCC tumors than their adjacent non-tumor counterparts. Statistically significant differences are denoted by asterisks above the bars, ***p < 0.0001, *p < 0.01.

**Fig. 2:**
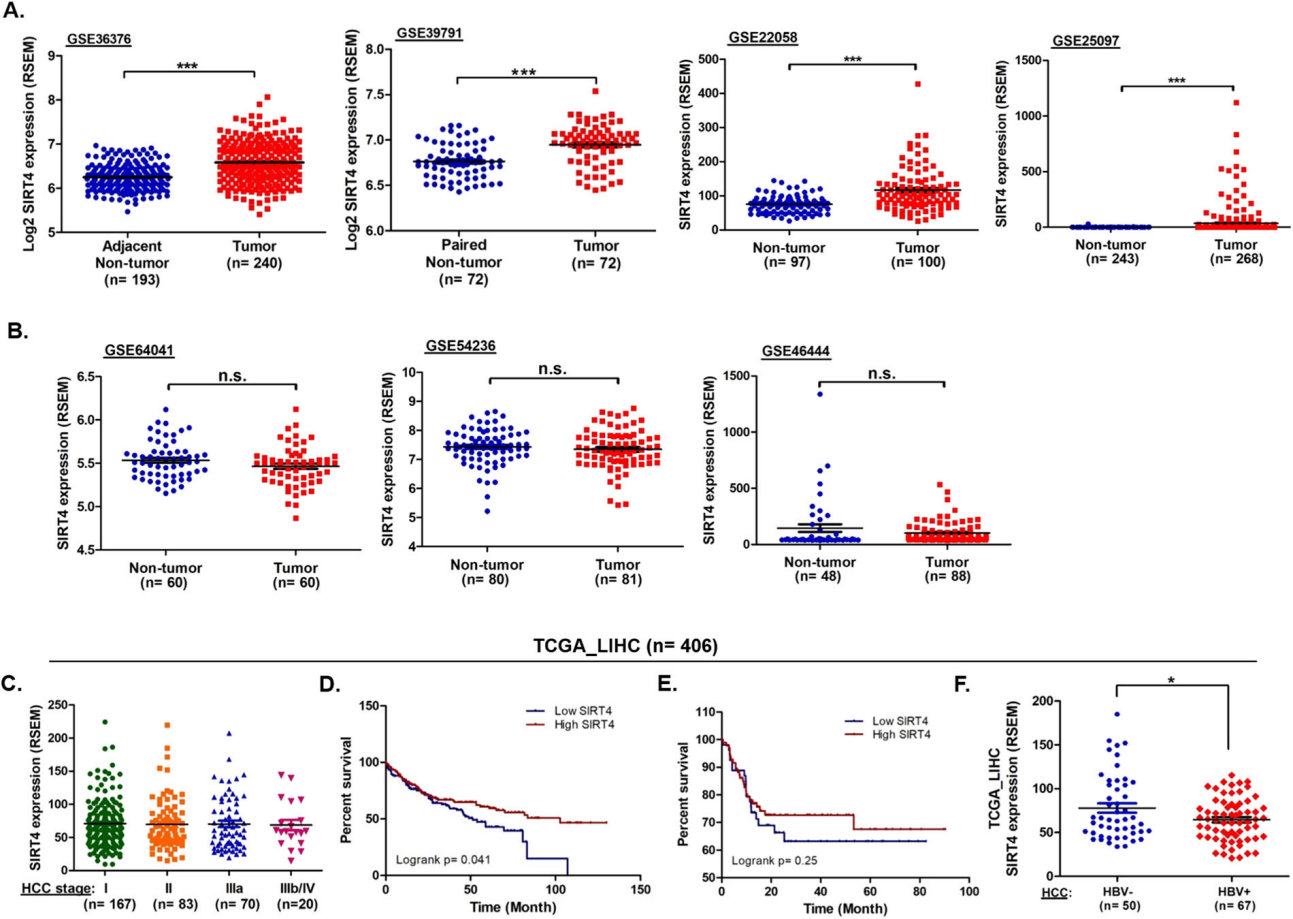
Validation of SIRT4 expression and its clinical relevance using NCBI GEO databases and TCGA liver database. **A** SIRT4 expression levels were significantly higher in tumor tissues than non-tumor tissues in GSE36376, GSE39791, GSE22058, and GSE25097 datasets. **B** SIRT4 expression levels were slightly higher in non-tumor tissues than tumor tissues in GS64041, GSE54236, and GSE46446 datasets. **C** Analysis of the TCGA_LIHC data on HCC tumors (*n* = 340) with disease TNM staging information stratified by the AJCC system. **D** Kaplan–Meier analysis of overall survival for 360 HCC patients with high and low SIRT4 expression. **E** Kaplan–Meier analysis of overall survival for 155 Asian HCC patients with high and low SIRT4 expression. **F** The correlation of the expression levels of SIRT4 with HBV infection status in Asian patients with HCC (***p < 0.0001, *p < 0.01; n.s.: not significant).

We examined the expression of SIRT4 in the 9 normal liver biopsies and 30 pairs of HCC tumors and their adjacent non-tumor counterparts. As shown in Fig. 1A, normal liver biopsies had a significantly higher SIRT4 mRNA expression than HCC tumor tissues and their peritumor tissues (both *p* < 0.0001). Intriguingly, the HCC tumor tissues had a higher SIRT4 expression than their non-tumor counterparts (Fig. 1A, B). In view of this, we analyzed SIRT4 expression using TCGA datasets retrieved from HCC patients. Of the seven NCBI GEO datasets studied, four datasets (GSE36376, GSE39791, GSE22058, GSE25097) had a higher SIRT4 expression in the HCC tumor than their adjacent non-tumor counterparts, while the other three datasets did not show any significant difference between the tumor and non-tumor tissues (Fig. 2A, B).

The TCGA_LIHC dataset is a well-characterized dataset that allows correlation between cancer phenotypes and genotypes. Among the 406 HCC patients in the TCGA_LIHC dataset, 360 cases had SIRT4 expression data available, and 340 of them had known TNM staging information stratified according to the American Joint Committee on Cancer (AJCC) system. As shown in Fig. 2C, no difference in SIRT4 expression was found between patients with different HCC TNM stages. Similarly, SIRT4 expression was not associated with TNM stages in our cohort of 30 HCC patients (*p* = 0.139 for SIRT4 expression in patients with HCC stage I/II vs. stage III).

The association between SIRT4 expression levels and patient survival was also investigated using data from the TCGA_LIHC dataset. The median expression level of SIRT4 for the 406 patients in the dataset was 61.14 Reads Per Kilobase Million (RPKM). HCC patients with SIRT4 expression >61.14 RPKM exhibited better survival than patients with low expression (<61.14 RPKM) of SIRT4 (Log-rank *p* = 0.041) (Fig. 2D). Subgroup analysis of the 155 Asian HCC patients in the dataset also showed that patients with high SIRT4 expression tended to have better overall survival than patients with low SIRT4 expression (Log-rank *p* = 0.25) (Fig. 2E), but the difference was not statistically significant.

### SIRT4 expression in HCC cell line models

**Fig. 3:**
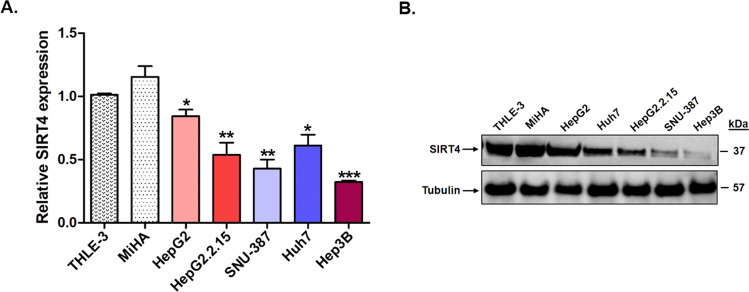
Downregulation of SIRT4 expression in liver cancer cell lines. **A** The expression of SIRT4 in five liver cancer cell lines relative to the expression in two normal liver cell lines THLE-3 and MiHA. **B** Protein expression of SIRT4 was downregulated in the liver cancer cell lines compared with normal liver cell lines. Statistically significant differences are denoted by asterisks above the bars, ***p < 0.0001, **p < 0.001, *p < 0.01.

In view of the above findings in HBV-infected patients with HCC, we also analyzed the expression of SIRT4 in a panel of human HCC cell lines. As shown in Fig. 3A, B, the two normal liver cell lines THLE3 and MiHA had a comparable SIRT4 expression at both the mRNA and protein levels. SIRT4 mRNA expression was significantly lower in all five HCC cell lines than in the two normal cell lines (*p* < 0.01 for HepG2 and Huh7; *p* < 0.001 for HepG2.2.15 and SNU-387; and *p* < 0.0001 for Hep3B) (Fig. 3A). The low expression of SIRT4 in the HCC cell lines was also confirmed at the protein level (Fig. 3B). Taken together, the reduced SIRT4 expression in the tumor/peritumor tissues and HCC cell lines compared with normal tissues and cell lines suggested a putative tumor-suppressive role of SIRT4 in HCC, and its downregulation was associated with poor patient survival.

### SIRT4 expression in HCC is associated with HBV infection

We next analyzed the correlation between SIRT4 expression and HBV infection. One hundred and seventeen patients in the TCGA_LIHC dataset had available data of HCC risk factors. Among them, 67 had HBV infection and 50 had other identifiable risk factors such as HCV infection and non-alcoholic fatty liver disease. As shown in Fig. 2F, the expression of SIRT4 in HCC patients with HBV infection was significantly lower than that in patients without HBV infection (*p* < 0.01). The association between HBV and SIRT4 was also observed in the HCC cell lines studied, in which the HCC cell lines harboring HBV (HepG2.2.15, SNU-387, and Hep3B) tended to have a lower expression of SIRT4 than those without HBV (HepG2 and Huh7) (Fig. 3A). Particularly, SIRT4 expression is lower in HepG2.2.15 than in the parental HepG2 cells (*p* < 0.05). The findings from both the TCGA_LIHC datasets and HCC cell lines suggested an association between SIRT4 expression and HBV infection.

### HBx induces cell growth and proliferation in HCC and suppressed SIRT4 expression

**Fig. 4:**
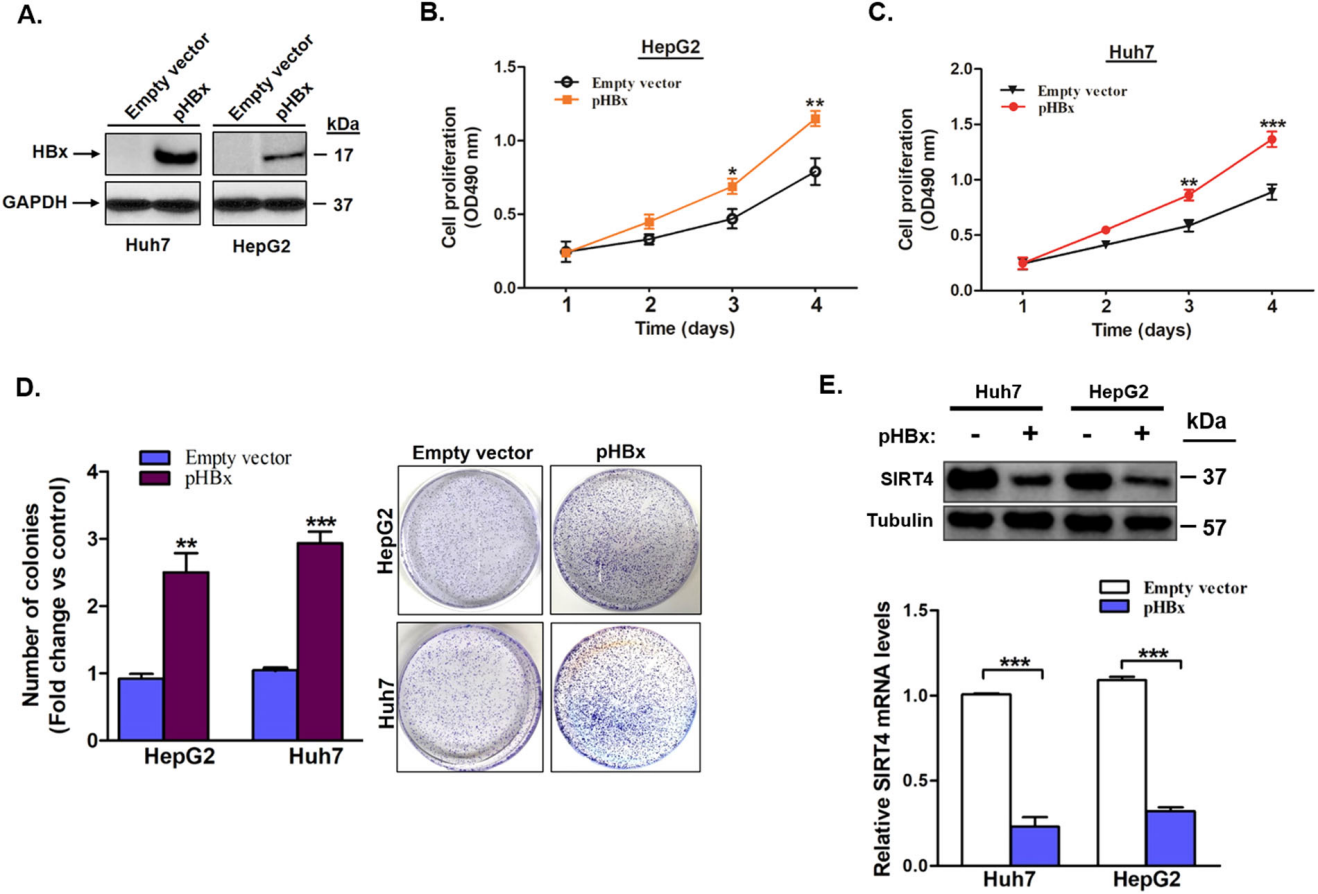
HBx transfection induces liver cancer cell growth and suppresses SIRT4 expression. **A** Western blot analysis confirms HBx protein expression in HBV negative cancer cell lines HepG2 and Huh7 transfected with HBx expression plasmid. **B** and **C** HBx transfection induces cell growth and proliferation in HepG2 and Huh7 cells. **D** HBx transfection induces colony formation in HepG2 and Huh7 cells transfected with HBx expression plasmid or control empty vector. The number of colonies was counted from five randomly selected views in cells transfected with HBx expression plasmid relative to the control cells transfected with empty vector. **E** qRT-PCR and Western blot analysis of SIRT4 expression levels in cells with stable HBx plasmid transfection. All error bars show the standard error of the mean (SEM). *p < 0.05, **p < 0.01, and ***p < 0.001.

Since HBx is the major tumorigenic protein of HBV, we investigated the association between HBx and SIRT4 expression in HCC. Using the HepG2 and Huh7 cells, we created two stably transfected, HBx-expressing cell lines, named HepG2-HBx and Huh7-HBx, respectively (Fig. 4A). We first confirmed the tumorigenic role of HBx in the two HBx stably transfected cell lines. Compared with cells transfected with the control plasmid, HepG2-HBx and Huh7-HBx had a significantly higher proliferation rate on both days 3 (*p* < 0.05 in HepG2 and *p* < 0.01 in Huh7) and day 4 (*p* < 0.01 in HepG2 and *p* < 0.001 in Huh7) (Fig. 4B, C). In addition, colony formation assay analysis showed that, compared with controls, both HepG2-HBx and Huh7-HBx displayed a significantly larger number of colonies (2.4-fold and 2.9-fold increases; *p* < 0.01 and *p* < 0.001, respectively) (Fig. 4D). These results indicated that HBx promoted uncontrolled cell growth and proliferation in the HBx-expressing cells. Next, we studied the effects of HBx on the expression levels of SIRT4. SIRT4 expressions in both Huh7-HBx and HepG2-HBx were significantly lower than that in the respective controls (both *p* < 0.001; Fig. 4E) and were comparable to the physiological expression levels in HCC patients with HBV infection (Supplementary Fig. 1). The reduced expression of SIRT4 in HBx-expressing cell lines suggested an interaction between HBx and SIRT4.

### Overexpression of SIRT4 induces cell apoptosis, cell-cycle arrest, and cell senescence

**Fig. 5:**
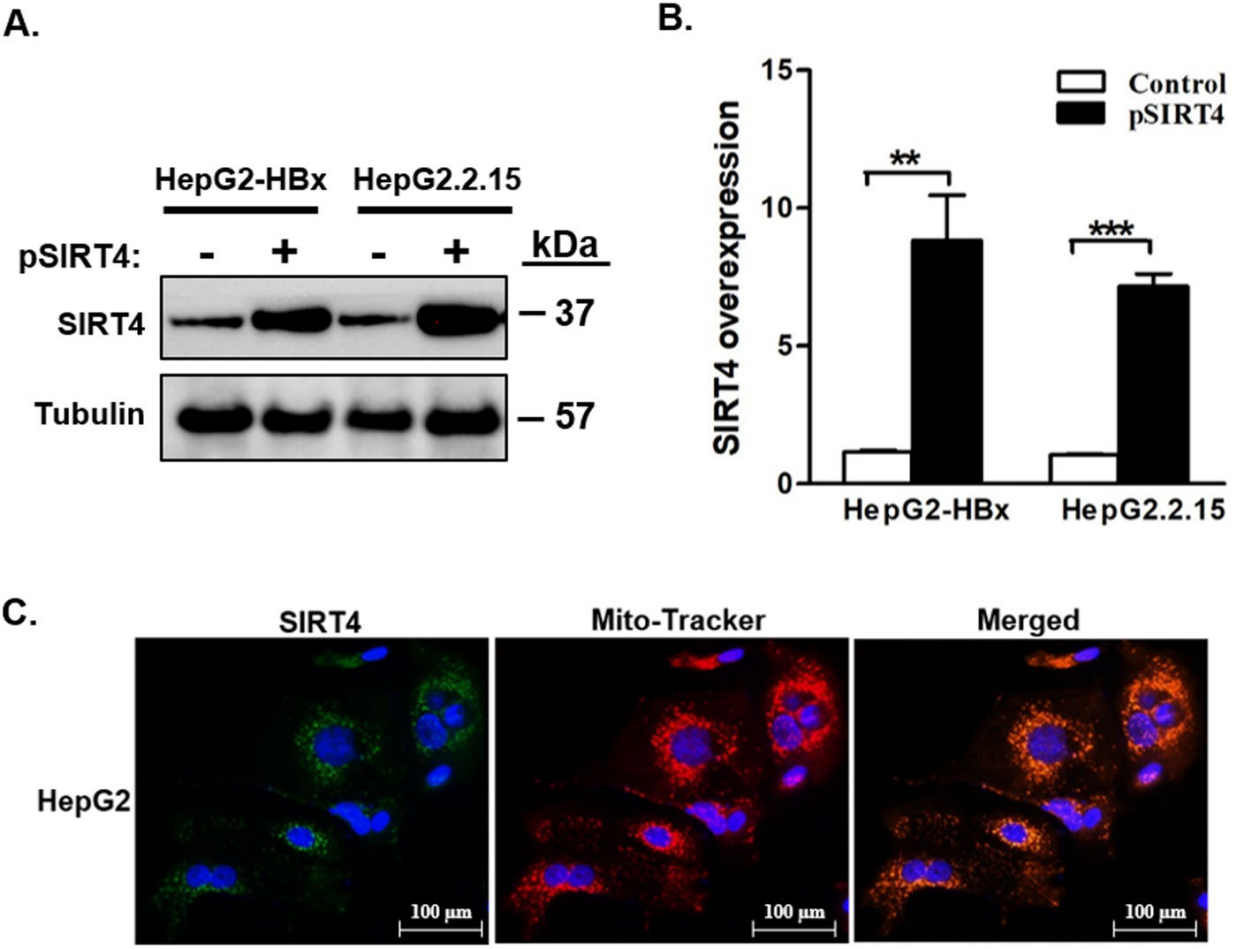
Overexpression of mitochondrial SIRT4 in liver cancer cell lines with stable HBx transfection. **A** and **B** Western blot and qRT-PCR confirm the overexpression of SIRT4 in HepG2-HBx and HepG2.2.15 cells after transfection of SIRT4 expression plasmid. **C** Fluorescent images showing mitochondrial localization of SIRT4. All error bars show the standard error of the mean (SEM). **p < 0.01 and ***p < 0.001.

We overexpressed SIRT4 in HepG2-HBx and its isogenic HBV-positive HepG2.2.15 cells, in which both had an HBx-positive background. Following ectopic SIRT4 plasmid transfection, overexpression of SIRT4 mRNA and protein was confirmed in both HepG2-HBx and HepG2.2.15 (Fig. 5A, B). Mitochondrial localization of ectopic SIRT4 expression was further confirmed by fluorescent microscopy (Fig. 5C).

**Fig. 6:**
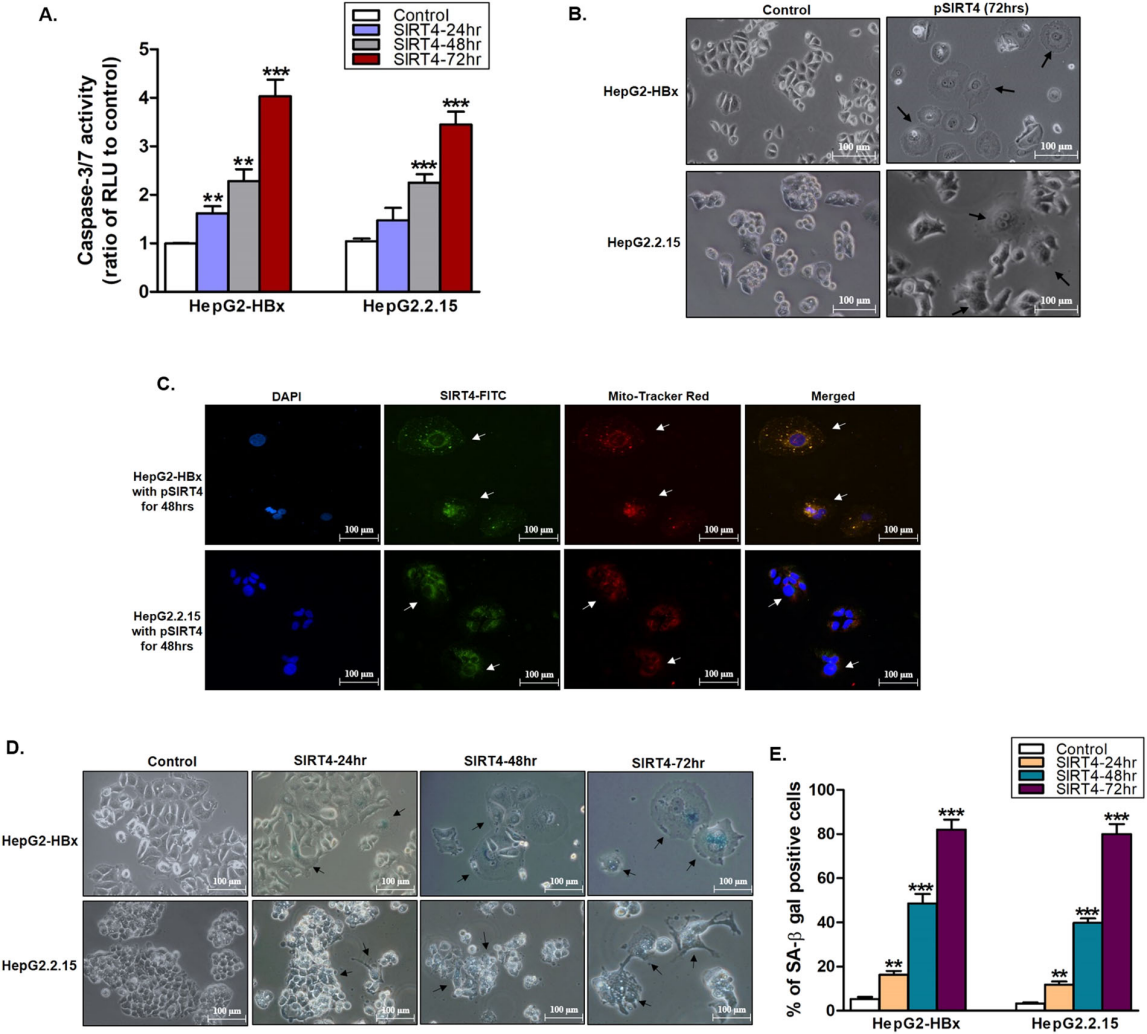
Overexpression of SIRT4 induces cell apoptosis, cell-cycle arrest, and cell senescence. **A** Increased caspase3/7 activity in cells after overexpression of SIRT4. Measurement of relative luminescence units (RLU) to control cells. **B** Representative black and white images showing SIRT4 overexpression induces senescence in HepG2-HBx and HepG2.215 cells with the morphological changes indicated by black arrows compared to their dividing cell counterparts. **C** Fluorescence staining of senescent cells with enlarged size and flattened shape as indicated by white arrows. **D** Representative images of SIRT4 overexpression cells show positive staining for SA-β-gal activity. Control cells show little or no staining. **E** The percentage of SA-β-gal positive cells were counted from five randomly selected views in cells transfected with SIRT4 expression plasmid relative to the control cells transfected with empty vector. Data are presented as mean ± SD of triplicate samples. **p < 0.001, and ***p < 0.0001.

The effects of SIRT4 overexpression were studied. As shown in Fig. 6A, SIRT4 overexpression significantly induced caspase 3/7 activity and hence apoptosis in a time-dependent manner in both HepG2-HBx (*p* < 0.001 at 24 and 48 h and *p* < 0.0001 at 72 h) and HepG2.2.15 (*p* < 0.0001 at 48 and 72 h) when compared with control cells. The induction of apoptosis by SIRT4 was demonstrated morphologically, where significant cell membrane blebbing was observed at 72 h post-transfection (Fig. 6B). Fluorescent staining of SIRT4-transfected HepG2-HBx and HepG2.2.15 cells showed that they became large, flat, and granular (Fig. 6C), and the majority of cells were oversized by 72 h post-transfection (Fig. 6B), suggesting that the SIRT4-overexpressing cells might have become senescent. To verify this, we measured the activity of β-galactosidase, a marker of cell senescence. As shown in Fig. 6D, E, β-galactosidase activity in the SIRT4-transfected HepG2-HBx and HepG2.2.15 increased with time. The percentage of β-galactosidasepositive cells significantly increased in the SIRT4overexpressing HepG2-HBx and HepG2.2.15 cells at 24 h (*p* < 0.001), 48 and 72 h (both *p* < 0.0001) when compared with controls. At 72 h post-transfection, the percentage of β–galactosidase positive cells was as high as 80% in both cell types, indicating that the majority of cells entered senescence upon SIRT4 overexpression.

### SIRT4 overexpression has anti-proliferative effects on liver cancer cells

**Fig. 7:**
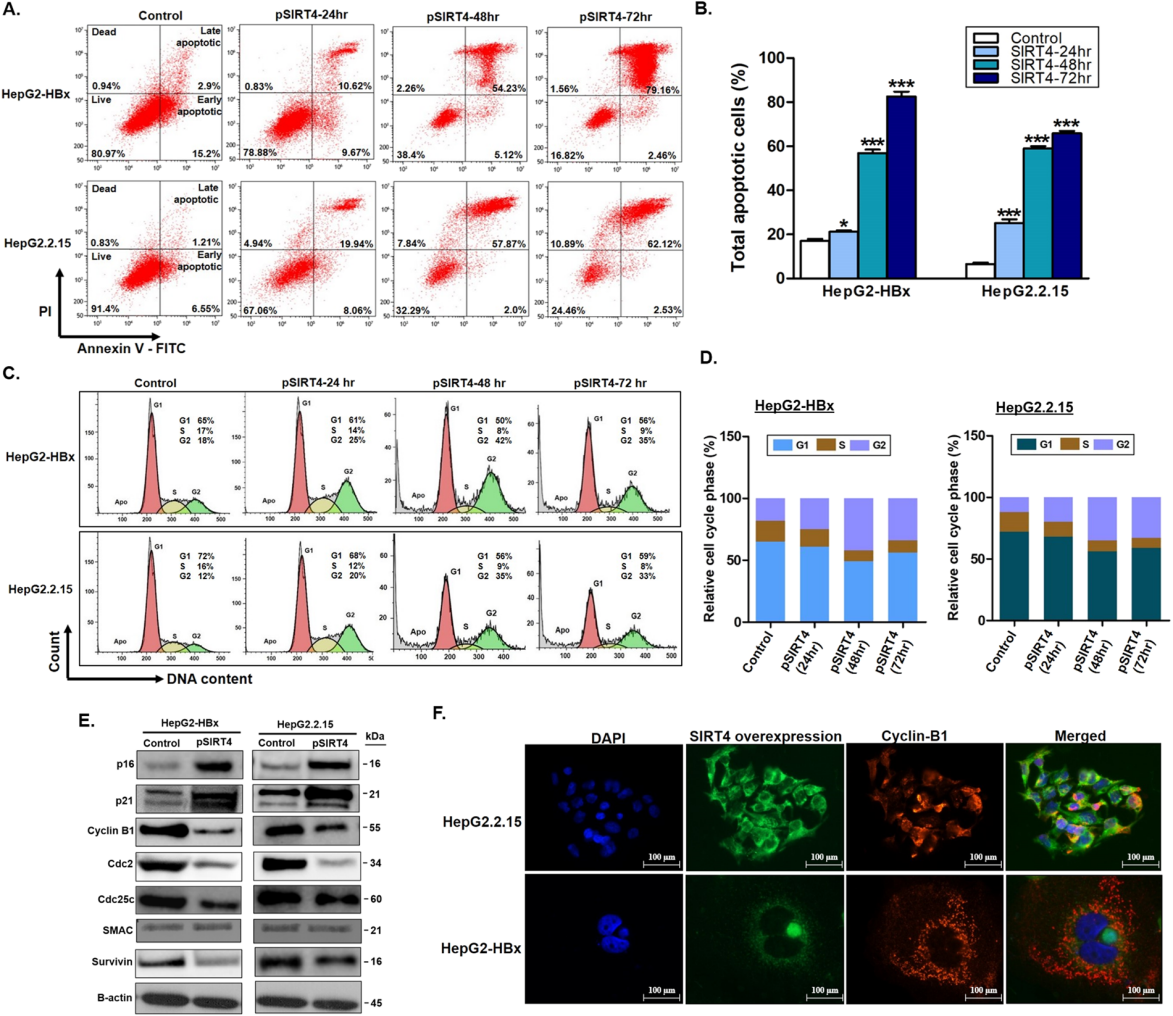
SIRT4 overexpression has anti-proliferative effects on liver cancer cells. **A** and **B** Flow cytometry analysis of HepG2-HBx and HepG2.2.15 cells after transfection with SIRT4 plasmid for 24, 48, and 72 h, followed by staining with annexin V-FITC and PI. The percentage of apoptotic cells was increased with time. **C** and **D** Flow cytometry analysis of cell cycle distribution indicating SIRT4 overexpression induces cell-cycle arrest at G2/M. **E** Western blot-analysis of cell-cycle-related proteins expression at 48 h after SIRT4 transfection. **F** Representative fluorescence images showing the retention of Cyclin B1 in the cytoplasm after SIRT4 overexpression. The quantitative data shown are mean ± SEM of triplicate samples. *p < 0.01 and ***p < 0.0001.

To study the anti-proliferative and cell-cycle regulatory function of SIRT4 in HCC, we assessed cell apoptosis and determined which cell cycle phase was specifically affected by flow cytometry. SIRT4 overexpression significantly induced cell apoptosis in a time-dependent manner in both HepG2-HBx and HepG2.2.15 cells but not in the control cells (Fig. 7A, B). In addition, SIRT4 overexpression enhanced G2/M phase cell cycle arrest and engaged the surviving cells in G2/M-arrest senescence (Fig. 7C, D). These observations were consistent with our findings on the activation of both caspase 3/7 (Fig. 6A) and β-galactosidase activity (Fig. 6E), indicating that SIRT4 overexpression in HBx-positive HCC cells conferred an anti-proliferative effect on the cells.

To mechanistically explore which cell cycle-related genes were involved in SIRT4-mediated G2/M arrest and induction of apoptosis, we assessed the expression of cell cycle checkpoint proteins p16, p21, Cyclin B1, Cdc2, Cdc25c, and mitochondrial apoptotic proteins SMAC and survivin. As shown in Fig. 7E, SIRT4 overexpression increased the expression of cell-cycle arrest proteins p16 and p21 and downregulated the cell-cycle progression proteins cyclin B1, Cdc2, and Cdc25c. For the mitochondrial apoptotic proteins, although an increased expression of the pro-apoptotic SMAC protein was not seen, a decreased expression of the anti-apoptotic survivin was observed in cells with SIRT4 overexpression (Fig. 7E). Fluorescence microscopy revealed that in cells with SIRT4 overexpression, there was cytoplasmic retention of cyclin B1, which prevented its translocation from the cytoplasm to the nucleus to execute cell cycle progression (Fig. 7F). These findings suggest that SIRT4 regulated G2/M arrest and apoptosis via modulation of the expression and subcellular locations of the cell regulatory proteins.

## Discussion

Among the SIRT family proteins, mitochondrial SIRT4 is the less well-understood protein^33,34^. The tumor-suppressive role of SIRT4 has recently been suggested in many types of tumors including HCC^26–30,32^. A previous report has demonstrated that suppression of SIRT4 expression promotes mitochondrial glutamine utilization to enhance HCC cell growth and HCC tumorigenesis, and knockout of SIRT4 promotes HCC lung metastasis in mice^32^. However, the role of SIRT4 in the development of HBV-related HCC and the association between SIRT4 and mitochondrial HBx is still unclear. In this study, we found that the expression of SIRT4 was significantly downregulated in HCC cell lines and in HCC tissues collected from patients with HBV-related HCC. Specifically, HCC cell lines harboring HBV showed a lower SIRT4 expression than cell lines without HBV. Further analysis on the TCGA liver datasets confirmed that SIRT4 expression in HCC tumors from patients with HBV infection was lower than that from patients without HBV. These findings suggested an association between HBV and SIRT4, and SIRT4 has a tumor-suppressive role in HBV-related HCC.

Our data further demonstrated that HBx suppressed mitochondrial SIRT4 expression, suggesting the existence of an interaction between HBx and SIRT4 in the development of HBV-related HCC. HBx, especially cytosolic HBx, is a well-known tumorigenic HBV protein with transactivating properties^35^. Our findings in the present study suggested a role of mitochondrial HBx in hepatocarcinogenesis via the suppression of SIRT4. Intriguingly, mitochondrial sirtuins have been reported to regulate essential metabolic pathways in response to viral infection^36^. We believe that the interaction of HBx and SIRT4 during viral infection (HBV infection in this case) and their roles on carcinogenesis deserve further studies, which may provide insights for the development of therapeutics against HBV-induced HCC.

Malignant proliferation and escape from apoptosis are two important pathological features of HCC^37–39^. To our knowledge, the present study is the first to demonstrate that mitochondrial SIRT4 protein regulates cell apoptosis and cell-cycle arrest at G2/M in HBV-related HCC. In addition to its apoptosis induction function via the suppression of the anti-apoptotic survivin and activation of caspase 3/7 activity, SIRT4 overexpression retains cyclin B1 in the cytoplasm and inhibits its translocation into the nucleus to drive the cell cycle progression. Cytoplasmic retention of cyclin B1 has been shown to maintain G2/M arrest and play a role in the regulation of cell cycle checkpoint and prevention of cancer progression^40,41^. Taken together, the present study suggested that one way of HBx-induced hepatocarcinogenesis is via SIRT4 suppression, resulting in the activation of survivin and nuclear translocation of cyclin B1, which in turn drives the cell cycle progression. This proposed axis of SIRT4-dependent inhibition of survivin and retention of cytoplasmic cyclin B1 in the suppression of tumor cell cycle progression and induction of apoptosis could shed light on the development of therapeutic targets against HCC tumorigenesis.

Cellular senescence is an intrinsic protective mechanism against cellular stress signals and it functions to eliminate damaged cells and prevent irreversible cell injuries^42^. Our findings of the role of SIRT4 in cellular senescence are consistent with previous reports which show that SIRT4 is upregulated in response to DNA damage stimuli, and SIRT4 overexpression can suppress cell proliferation in mouse embryonic fibroblasts^43^. Activation of SIRT4 can induce G2/M arrest in HCC cells, suggesting that targeting on cellular senescence pathways may shed light for future development of a “pro-senescence” therapy for clinical applications. Important questions such as how SIRT4 induces senescence and the cross-talk between SIRT4-induced cell senescence and apoptosis deserve further studies.

Nevertheless, the detailed mechanisms by which silencing of SIRT4 initiates HCC remains to be studied. While data from our patient cohort showed a higher SIRT4 expression in the tumors than their adjacent non-tumors, data from the NCBI and TCGA databases were inconclusive. We speculate that the low SIRT4 expression in the non-tumors may be due to its possible role in the initiation of HCC. In many cancers, SIRT4 downregulation has been observed at the early stages of tumors^30,32,44^. In the present study, the majority (83.3%) of patients had liver fibrosis and/or cirrhosis, which are regarded as preneoplastic conditions. Thus, the downregulation of SIRT4 in these early preneoplastic conditions may contribute further to the development of HCC. Nonetheless, the role of SIRT4 in hepatocarcinogenesis, as well as its dynamic changes in the expression during the process, requires further analysis using animal models and human preneoplastic liver tissues.

In summary, our study demonstrated that HBV infection, particularly HBx expression, interfered with mitochondrial SIRT4 expression. The oncogenic HBx protein is associated with the downregulation of SIRT4. Activation of SIRT4 could induce cell apoptosis and G2/M arrest, thereby halting cancer cell cycle progression. Taken together, our findings suggest that the tumor-suppressive role of SIRT4 is downplayed by HBx expression, which may be one of the tumorigenic properties of HBx. A further in-depth study on the intrinsic molecular mechanism of SIRT4, as well as its interaction with HBx, might offer potential targeted genes for molecular therapy in HCC.

## Materials and methods

### Tissue samples

Thirty tumor tissues and their adjacent non-tumor liver tissues were collected from chronic hepatitis B patients who subsequently developed HCC and had surgical resection at the Queen Mary Hospital, Hong Kong. Liver biopsies obtained from 9 patients without HCC and HBV-infection were included as controls. The tissues were immediately snap-frozen in liquid nitrogen and stored at −80 °C until analysis. Written informed consent was obtained from all individuals. This study was approved by the Institutional Review Board, Hong Kong Hospital Authority West Cluster, and The University of Hong Kong (UW19-747).

### Cell lines and culture conditions

Human cancer cell lines (SNU-387, Huh7, HepG2, HepG2.2.15, and Hep3B) and a normal liver cell line THLE-3 were acquired from the American Type Culture Collection (ATCC; Manassas, VA, USA). Another normal liver cell line MiHA was obtained from the Shanghai Institutes for Biological Sciences, Chinese Academy of Sciences. Cells were cultured in medium suggested by the providers or in RPMI-1640 medium with 10% fetal bovine serum (Thermo Fisher Scientific, Waltham, MA, USA) in a humidified incubator with 5% CO_2_ at 37 °C.

### Nuclear acids and protein extraction

Nucleic acids and proteins were extracted from liver tissues using the QIAamp Allprep Kit (Qiagen, Hilden, Germany) following the manufacturer’s instructions. Total RNA was extracted from cell lines using TRIzol reagent (Thermo Fisher Scientific) according to the manufacturer’s instructions. The quantity and quality of the nucleic acids were assessed using the NanoDrop 2000 Spectrophotometer (Thermo Fisher Scientific).

### Real-time PCR analysis of gene expression

cDNA was synthesized using the First Strand cDNA synthesis kit for RT-PCR (Roche Diagnostics, Mannheim, Germany). Gene expression was measured by the SYBR Green PCR master mix (Roche Diagnostics). Gene expression levels were normalized with GAPDH as an internal control using the 2^−∆ΔCT^ method. Primer sequences for PCR amplification were listed in Supplementary Table S1.

### Gene expression and survival analysis using TCGA datasets

The expression of SIRT4 in HCC was analyzed using data obtained from The Cancer Genome Atlas Liver HCC (TCGA_LIHC) project (https://www.cancer.gov/tcga) and NCBI GEO databases (Accession Nos. GSE39791, GSE22058, GSE25097, GSE36376, GSE46444, GSE54236, and GSE64041)^45^. cBioPortal for Cancer Genomics website (http://www.cbioportal.org/public-portal/) was used to access TCGA mRNA expression and clinical datasets. The effect of differential gene expression on patient survival was classified into high and low expression groups using the median level as a reference.

### Plasmid and transfection

The vectors (pcDNA3.1 and pCR-Blunt II-TOPO) were purchased from Thermo Fisher Scientific. The HBx expression plasmid (pHBx) was constructed as previously reported^46^. SIRT4 plasmid provided by Mammalian Gene Collection (MGC:130046) was obtained from PlasmID Repository at Harvard Medical School. Cells were seeded in culture discs or plates with low serum medium to ~80% confluency at the time of transfection. Transfection of HBx and SIRT4 plasmids was performed using the Lipofectamine 3000 transfection reagent (Thermo Fisher Scientific). HepG2 and Huh7 cells with stable HBx transfection were selected by Geneticin (300 µg/ml) (Thermo Fisher Scientific) for 14 days before use for analysis. Stable expression of HBx was verified by mRNA and protein analyses.

### Western Blot analysis

Cellular proteins were extracted using the Mammalian Cell Lysis Reagent (Thermo Fisher Scientific). Protein concentration was determined by the Bradford protein assay kit (Bio-Rad, Hercules, CA, USA). Equal amounts of total protein were loaded and run on 10% SDS–polyacrylamide gels, followed by transferring proteins to PVDF membranes. The membranes were blocked with 5% milk and probed with relevant primary antibodies against HBx (Ab2741) (Abcam, Cambridge, UK), p16 (CS18769), p21 (CS2947), Cyclin B1 (CS4138), Cdc2 (CS29114), Cdc25c (CS9528), SMAC (CS15108), survivin (CS2808), tubulin (CS2125), β-actin (CS3700) (Cell Signaling, Danvers, MA, USA), or SIRT4 (PA572970) (Thermo Fisher Scientific) overnight at 4 °C. Protein expression was detected using the ECL detection system (GE Healthcare, Chicago, IL, USA). Intensities of protein bands were quantified using the Image J software (NIH, Bethesda, MD, USA).

### Cell proliferation assay

HepG2 and Huh7 cells (~1 × 10^4^) were seeded in 96-well plates. Cell proliferation was determined by using the CellTiter 96^®^ Aqueous One solution (Promega, Madison, MI, USA) according to the manufacturer’s protocol. The optical density value of each well was measured at 24, 48, 72, and 96 h at 490 nm using the CLARIOstar Plus microplate reader (BMG Labtech, Ortenberg, Germany). Experiments were performed in triplicate.

### Caspase3/7 activity

Caspase 3/7 activity was analyzed using the Caspase-GLO 3/7 kit (Promega) following the manufacturer’s protocol. Approximately 1 × 10^6^ stable HBx transfected HepG2 cells and its isogenic HBV positive HepG2.2.15 cells were transiently transfected with SIRT4 plasmid. At 24, 48, and 72 h post-transfection, cells were incubated with medium supplemented with 100 μl caspase-3/7 reagent for 3 h at room temperature. Luminescence released from cleaved caspase substrate was measured using the CLARIOstar Plus microplate reader (BMG Labtech). The resulting data were expressed as OD values and normalized to control cells.

### Cell cycle and cell apoptosis analysis

Cell apoptosis was performed using the FITC Annexin V Apoptosis Detection Kit (BD Biosciences) following the manufacturer’s instructions. For cell cycle analysis, cells were fixed in 70% ice-cold ethanol for 2 h at 4 °C and treated with propidium iodide (PI) solution containing 50 µg/ml RNase A (Thermo Fisher Scientific). Cell apoptosis, DNA content, and cell cycle distribution were analyzed by the CytoFLEX flow cytometer (Beckman Coulter, Brea, CA, USA). Results were calculated and presented by Kaluza analysis software (Beckman Coulter).

### Senescence-associated beta-galactosidase assay

Cell senescence was determined using the senescence β-galactosidase (SA-β-gal) staining kit (Cell Signaling) following the manufacturer’s instructions. At 24, 48, and 72 h post-transfection of SIRT4 plasmid, cells were fixed with a fixative solution for 15 min, followed by staining with β-gal solution at 37 °C overnight in a dry incubator. Cells were checked under a phase-contrast microscope (×200 magnification) for the development of blue color.

### Immunofluorescence

Cells were cultured in chamber slides with or without SIRT4 overexpression. The attached cells were fixed with 4% paraformaldehyde and incubated with primary antibodies at 4 °C overnight. Then the cells were washed and incubated with appropriate secondary antibodies for 1 h at room temperature and counterstained the nucleus with 4′,6-diamidino-2-phenylindole (DAPI) (Thermo Fisher Scientific). Fluorescent images were captured using a fluorescent microscope (Carl Zeiss, Oberkochen, Germany).

### Statistical analysis

Continuous variables were expressed as mean ± standard error of the mean (SEM) and analyzed using the Student’s *t*-test. Survival analysis was performed by Kaplan–Meier method, and survival between groups was estimated by the log-rank test. All statistical analysis was performed using GraphPad Prism 5.0 (GraphPad Software, Inc., San Diego, CA). A *p* value of <0.05 was considered statistically significant.

## Supplementary information

Supplementary figure legends

Supplementary Table

Supplementary Figure

## Data Availability

The datasets used and/or analyzed during the current study are available from the corresponding author on reasonable request.
