# Supplementary material for: Tumor suppressive role of mitochondrial sirtuin 4 in induction of G2/M cell cycle arrest and apoptosis in hepatitis B virus-related hepatocellular carcinoma
Source: Cell Death Discov. 2021 Apr 30;7:88. doi: 10.1038/s41420-021-00470-8 (PMC8087836; doi:10.1038/s41420-021-00470-8)
Supplement: Supplementary file 1 — Supplementary figure legends [file 41420_2021_470_MOESM1_ESM.docx]

**Tumor suppressive role of mitochondrial sirtuin 4 in induction of G2/M cell cycle arrest and apoptosis in hepatitis B virus-related hepatocellular carcinoma**

Fung-Yu Huang,^1*^ Danny Ka-Ho Wong,^1,2*^ Wai-Kay Seto,^1,2^ Lung-Yi Mak,^1,2^ Tan-To Cheung,^2,3^ Man-Fung Yuen^1,2**^

**Supplementary information**

Table S1: Primers sequence used in this study

Table S2. Clinical characteristics of patients with hepatocellular carcinoma

**Supplementary Figure 1.** Protein expression analysis of SIRT4 in HCC tissues and cell lines. (A) Western blot analysis showing comparable protein expression of SIRT4 in human HCC tissues (D87 and D93) and cell lines (Huh7-HBx and HepG2-HBx) with stable transfection of HBx plasmid.
