# Supplementary material for: Tumor suppressive role of mitochondrial sirtuin 4 in induction of G2/M cell cycle arrest and apoptosis in hepatitis B virus-related hepatocellular carcinoma
Source: Cell Death Discov. 2021 Apr 30;7:88. doi: 10.1038/s41420-021-00470-8 (PMC8087836; doi:10.1038/s41420-021-00470-8)
Supplement: Supplementary file 2 — Supplementary Table [file 41420_2021_470_MOESM2_ESM.docx]

Table S1: Primers sequence used in this study

| Name | Sequences (5’ – 3’) | Annealing temperature (°C) |
| --- | --- | --- |
| HBx-1548F | 5’-CTCCCCGTCTGTGCCTTCT-3’ |  |
| HBx-1823B | 5’-AGATGATTAGGCAGAGGTGAAAAA-3’ | 60 |
| SIRT4-F | 5’- TCGTTTTCTTCGGGGACACA-3’ |  |
| SIRT4-B | 5;- CAACTCTCCACAACGAGAAT-3’ | 60 |
| GAPDH-F | 5’-CAAATTCCATGGCACCGTCA-3’ |  |
| GAPDH-B | 5’-TCTCGCTCCTGGAAGATGGTGA-3’ | 60 |

Table S2. Clinical characteristics of patients with hepatocellular carcinoma

| **Groups** | **Number of cases (%)** |
| --- | --- |
| Age |  |
| ＜50 | 10(33.3) |
| ≥50 | 20(66.7) |
| Gender |  |
| Male | 24(80) |
| Female | 6(20) |
| TNM stage |  |
| Ⅰ | 7(23.3) |
| Ⅱ | 14(46.7) |
| Ⅲ | 9(30) |
| Tumor differentiation |  |
| Well | 10(33.3) |
| Moderate | 15(50) |
| Poor | 5(16.7) |
| Fibrosis/cirrhosis in adjacent non-tumor |  |
| Yes | 25(83.3) |
| No | 5(16.7) |
| Tumor invasion |  |
| Yes | 16(53.3) |
| No | 14(46.7) |
| Tumor encapsulation |  |
| Absent | 10(33.3) |
| Present | 20(66.7) |
| Tumor size (cm) |  |
| ≤5 | 9(30) |
| >5 | 21(70) |
