# Supplementary figures and images for: Tumor suppressive role of mitochondrial sirtuin 4 in induction of G2/M cell cycle arrest and apoptosis in hepatitis B virus-related hepatocellular carcinoma
Source: Cell Death Discov. 2021 Apr 30;7:88. doi: 10.1038/s41420-021-00470-8 (PMC8087836; doi:10.1038/s41420-021-00470-8)

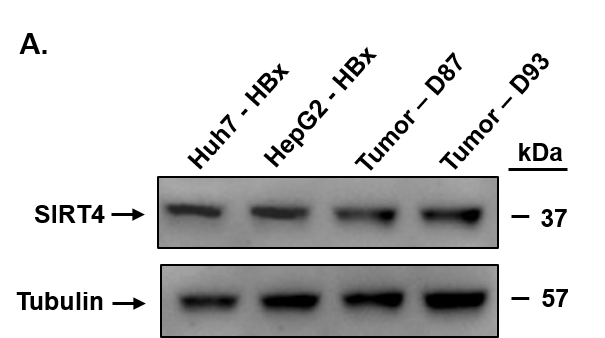

Supplement: Supplementary file 3 — Supplementary Figure [file 41420_2021_470_MOESM3_ESM.tif]
